# ROLE CAPTIVITY, DAILY SOCIAL ISOLATION, AND THE MENTAL HEALTH OF DEMENTIA CAREGIVERS

**DOI:** 10.1093/geroni/igad104.1505

**Published:** 2023-12-21

**Authors:** Frank Puga, Danny Wang, Natashia Bibriescas, Abigail Poe, Loreli Alvarez, Rita Jablonski, David Vance

**Affiliations:** University of Alabama at Birmingham, Birmingham, Alabama, United States; The Pennsylvania State University, University Park, Pennsylvania, United States; University of Texas at Austin, Austin, Texas, United States; University of Alabama at Birmingham, Birmingham, Alabama, United States; The University of Alabama at Birmingham, Birmingham, Alabama, United States; University of Alabama at Birmingham, Birmingham, Alabama, United States; University of Alabama at Birmingham, Birmingham, Alabama, United States

## Abstract

Family members providing informal care to individuals living with Alzheimer’s disease and related dementias (ADRDs) have an increased risk of poor mental and cognitive health. Social connection has been proposed as an intervention target to support the health and well-being of ADRD caregivers; however, little is known about daily variation in social connection, contextual factors that impact social connection, and its association with caregiver mental health experiences. Thus, the purpose of this study was to examine the relationship between role captivity and daily social isolation (i.e., lack of social connection) among dementia caregivers, as well as associations between social isolation and depression and anxiety-related symptoms. A sample of community-dwelling ADRD caregivers (N=30) completed a baseline survey followed by 14 days of daily diaries (n=323 data points). Participants were asked about role captivity, social isolation, and daily depression and anxiety-related symptoms. Data were analyzed using mixed-level modeling. Role captivity reported at baseline was significantly associated with daily social isolation (B=0.82, 95% CI [0.68, 0.96], p <0.001). Further, daily social isolation was significantly associated with both depression (B=0.69, 95% CI [0.61, 0.77], p <0.001) and anxiety (B=0.55, 95% CI [0.48, 0.62], p <0.001) related symptoms reported on a given day. The findings from this study contribute knowledge on ecologically valid interventions that target daily experiences of social isolation and support the mental health of dementia caregivers. Such interventions are critically important given the proposed links between mental health, social isolation, and cognitive health in later life.

